# Supplementary material for: Pyrocatalysis—The DCF assay as a pH-robust tool to determine the oxidation capability of thermally excited pyroelectric powders
Source: PLoS One. 2020 Feb 6;15(2):e0228644. doi: 10.1371/journal.pone.0228644 (PMC7004307; doi:10.1371/journal.pone.0228644)
Supplement: S1 Fig — (a) 1–10 nM, (b) 10–150 nM, (c) 150–1000 nM. (PDF) [file pone.0228644.s001.pdf]

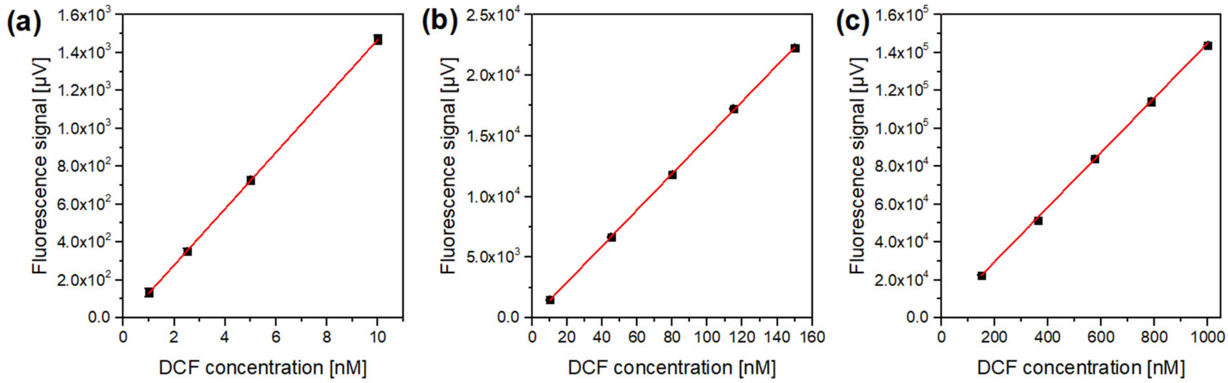

Figure S1. Results and linear regression of external calibration of DCF with fluorescence spectroscopy in three concentration ranges: (a) 1 – 10 nM, (b) 10 – 150 nM, (c) 150 – 1000 nM.
