# Supplementary material for: Pyrocatalysis—The DCF assay as a pH-robust tool to determine the oxidation capability of thermally excited pyroelectric powders
Source: PLoS One. 2020 Feb 6;15(2):e0228644. doi: 10.1371/journal.pone.0228644 (PMC7004307; doi:10.1371/journal.pone.0228644)
Supplement: S2 Fig — In a typical experiment, 12 micro tubes were placed in every second slot (numbered slots). (PDF) [file pone.0228644.s002.pdf]

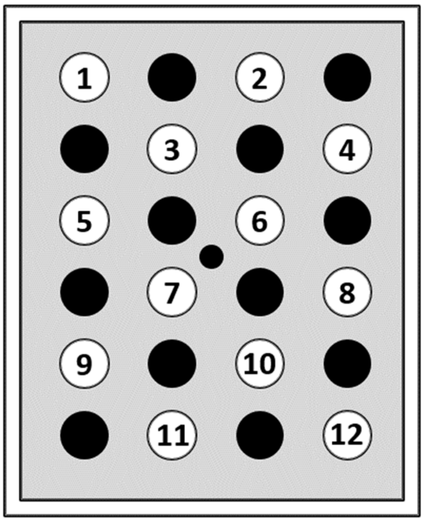

Figure S1. Schematic sketch of the aluminium block of the thermomixer with 24 slots. In a typical experiment, 12 micro tubes were placed in every second slot (numbered slots).
