# Supplementary material for: Pyrocatalysis—The DCF assay as a pH-robust tool to determine the oxidation capability of thermally excited pyroelectric powders
Source: PLoS One. 2020 Feb 6;15(2):e0228644. doi: 10.1371/journal.pone.0228644 (PMC7004307; doi:10.1371/journal.pone.0228644)
Supplement: S3 Fig — It consists of a 5 min equilibration phase at 32.5 °C followed by nine full cycles and a 5 min cooling phase back to the starting temperature. (PDF) [file pone.0228644.s003.pdf]

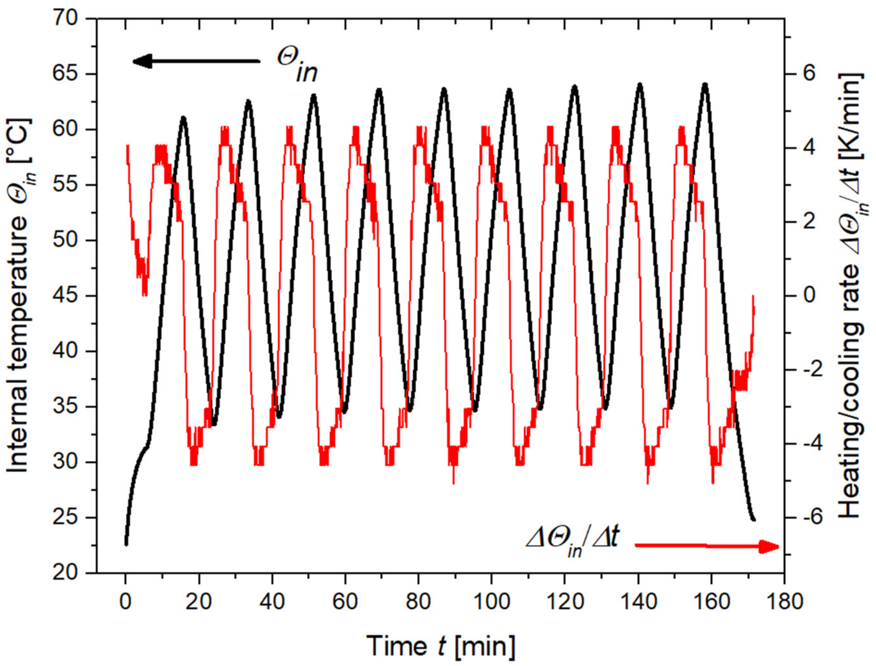

Figure S1. Measured temperature inside the reaction vessel Θ_{in} over time t and corresponding heating/cooling rate $\Delta\Theta_{in}/\Delta t$ for the whole temperature program used in the DCHF-oxidation experiments. It consists of a 5 min equilibration phase at 32.5 °C followed by nine full cycles and a 5 min cooling phase back to the starting temperature.
