# Supplementary material for: Pyrocatalysis—The DCF assay as a pH-robust tool to determine the oxidation capability of thermally excited pyroelectric powders
Source: PLoS One. 2020 Feb 6;15(2):e0228644. doi: 10.1371/journal.pone.0228644 (PMC7004307; doi:10.1371/journal.pone.0228644)
Supplement: S4 Fig — (PDF) [file pone.0228644.s004.pdf]

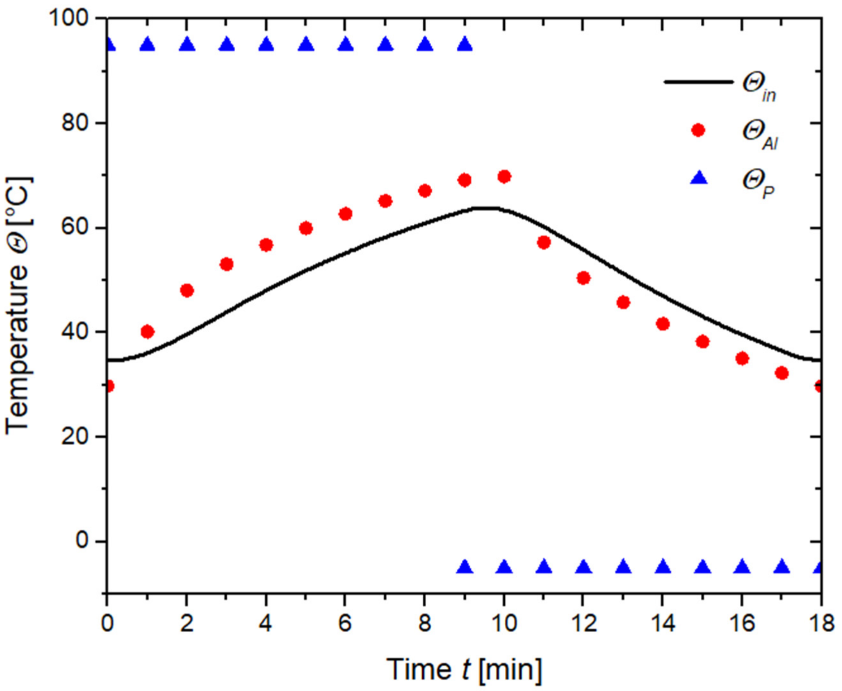

Figure S1. Measured temperature inside the reaction vessel Θ_{in}, of the aluminium block of the thermoshaker Θ_{Al} and the preset temperature Θ_P over time t for one temperature cycle used in the DCHF-oxidation experiments.
