# Supplementary material for: Pyrocatalysis—The DCF assay as a pH-robust tool to determine the oxidation capability of thermally excited pyroelectric powders
Source: PLoS One. 2020 Feb 6;15(2):e0228644. doi: 10.1371/journal.pone.0228644 (PMC7004307; doi:10.1371/journal.pone.0228644)
Supplement: S5 Fig — Inset shows composition of 002/020 reflection with respect to cubic and tetragonal fraction. (PDF) [file pone.0228644.s005.pdf]

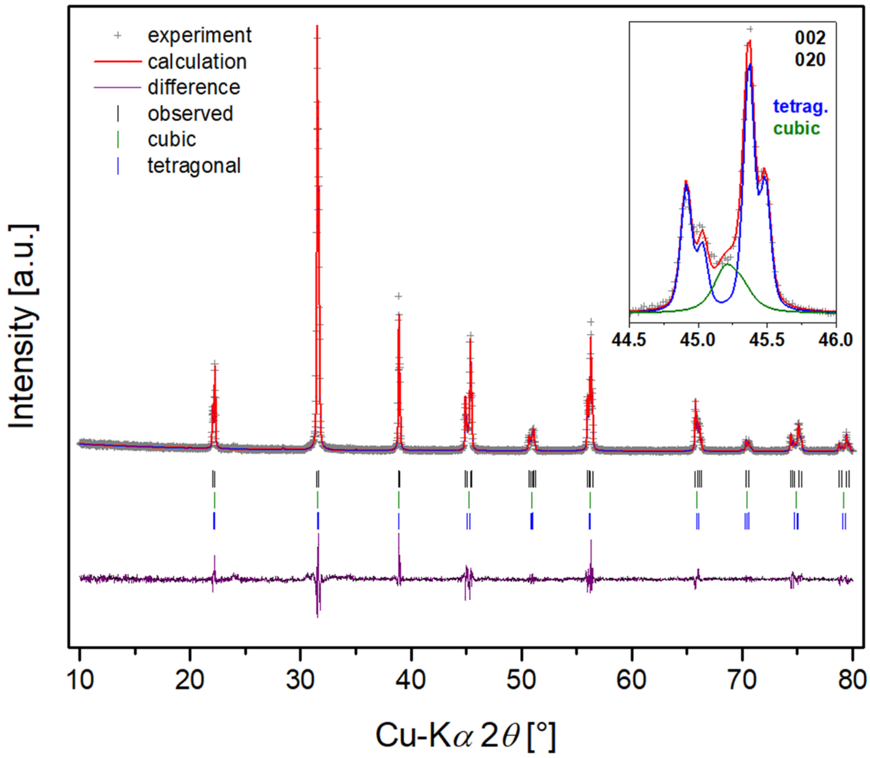

Figure S1. XRD diffraction Rietveld refinement results of $BaTiO_3$ powder. Inset shows composition of 002/020 reflection with respect to cubic and tetragonal fraction.
