# Supplementary figures and images for: Pyrocatalysis—The DCF assay as a pH-robust tool to determine the oxidation capability of thermally excited pyroelectric powders
Source: PLoS One. 2020 Feb 6;15(2):e0228644. doi: 10.1371/journal.pone.0228644 (PMC7004307; doi:10.1371/journal.pone.0228644)

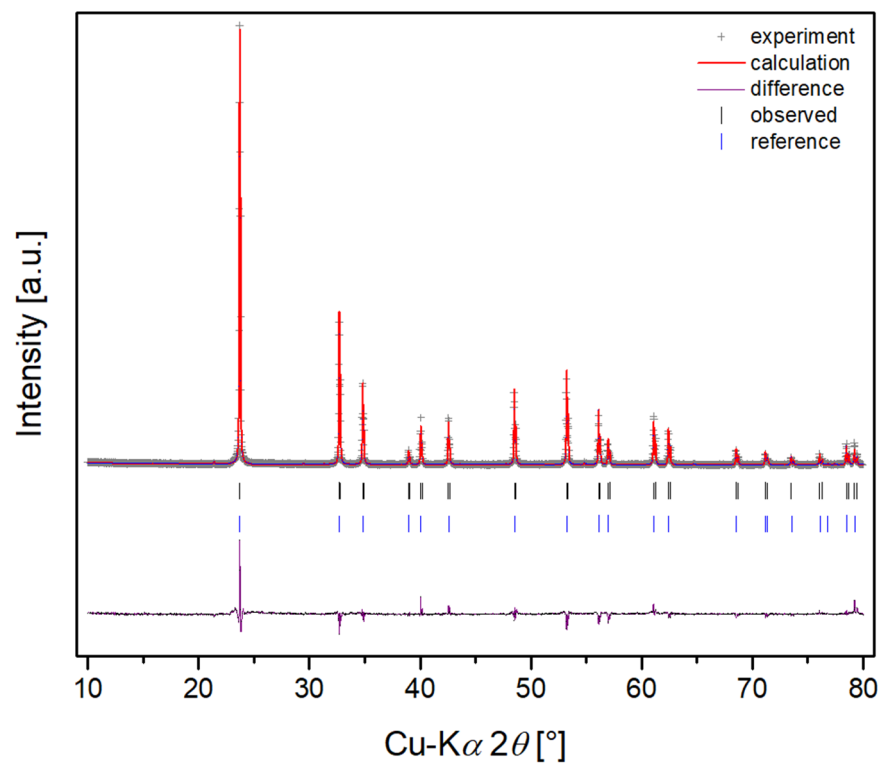

**Figure S1.** XRD diffraction Rietveld refinement results of  $\text{LiNbO}_3$  powder.

Supplement: S6 Fig — (PDF) [file pone.0228644.s006.pdf]
