# Supplementary material for: Pyrocatalysis—The DCF assay as a pH-robust tool to determine the oxidation capability of thermally excited pyroelectric powders
Source: PLoS One. 2020 Feb 6;15(2):e0228644. doi: 10.1371/journal.pone.0228644 (PMC7004307; doi:10.1371/journal.pone.0228644)
Supplement: S7 Fig — (PDF) [file pone.0228644.s007.pdf]

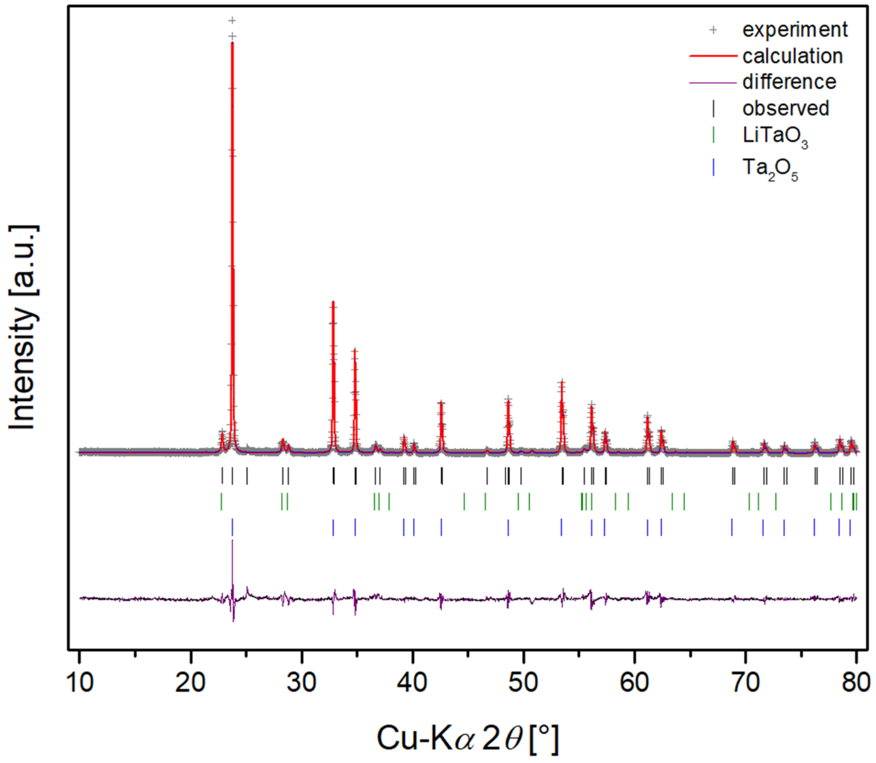

Figure S1. XRD diffraction Rietveld refinement results of $LiTaO_3$ powder with 11% Ta_2O_5 content.
