# Supplementary material for: Pyrocatalysis—The DCF assay as a pH-robust tool to determine the oxidation capability of thermally excited pyroelectric powders
Source: PLoS One. 2020 Feb 6;15(2):e0228644. doi: 10.1371/journal.pone.0228644 (PMC7004307; doi:10.1371/journal.pone.0228644)
Supplement: S8 Fig — Inset plot: Relative intensity of the DCF solution at different pH values. (PDF) [file pone.0228644.s008.pdf]

For the comparison of the fluorescence emission and excitation spectra of DCF at different pH values 800 µL of a 150 nM DCF solution were diluted with 2500 µL water, NaOH (0.01/ 0.1/ 1/ 10 mM) or HCl (0.01/ 0.1/ 1/ 10/ 40 mM) solutions. Additionally, the final pH of the DCF solutions was measured.

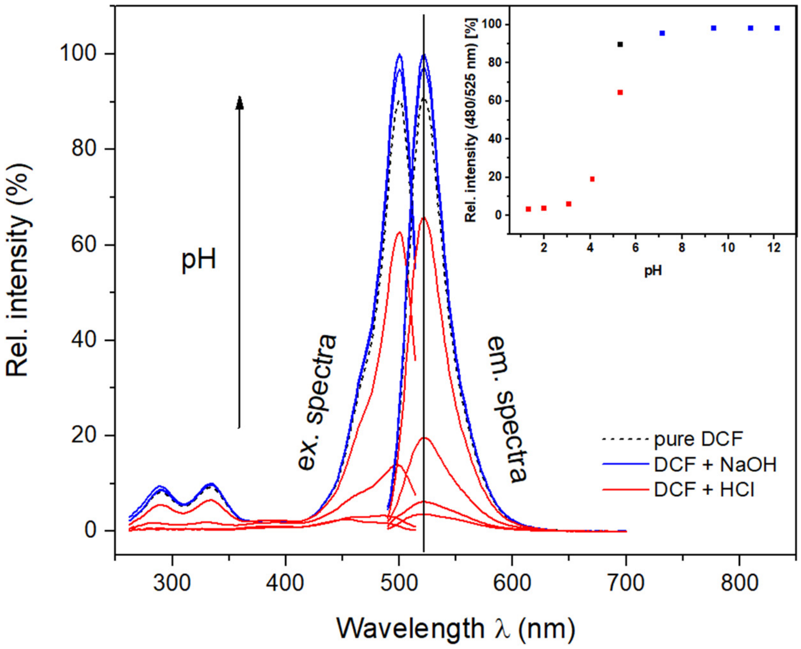

Figure S1. Fluorescence excitation and emission spectra of a DCF solution diluted with water or different concentrations of NaOH or HCl (λ_{ex} = 480 nm; λ_{em} = 525 nm) for pH adjustment. Inset plot: Relative intensity of the DCF solution at different pH values.
