# Supplementary material for: Pyrocatalysis—The DCF assay as a pH-robust tool to determine the oxidation capability of thermally excited pyroelectric powders
Source: PLoS One. 2020 Feb 6;15(2):e0228644. doi: 10.1371/journal.pone.0228644 (PMC7004307; doi:10.1371/journal.pone.0228644)
Supplement: S9 Fig — DCHF: dichlorodihydrofluorescein, DA: diacetate. (PDF) [file pone.0228644.s009.pdf]

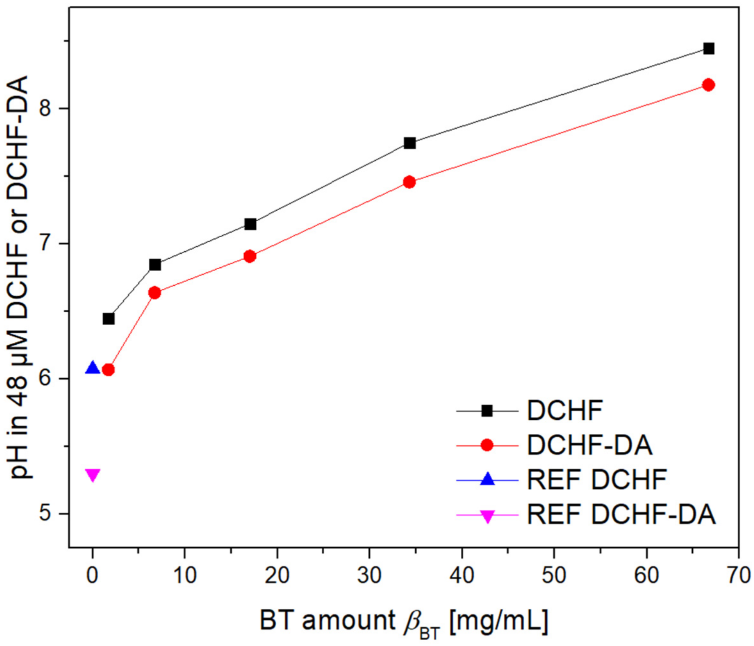

Figure S1. Comparison of the pH-increase with the DCHF-DA- and DCHF-based reaction protocols (c_0 = 48 µM) of the DCF-assay for increasing amounts β_{BT} of $BaTiO_3$ powder (BT) and reference experiments (REF) after thermal treatment. DCHF: dichlorodihydrofluorescein, DA: diacetate.
