# Supplementary material for: Pyrocatalysis—The DCF assay as a pH-robust tool to determine the oxidation capability of thermally excited pyroelectric powders
Source: PLoS One. 2020 Feb 6;15(2):e0228644. doi: 10.1371/journal.pone.0228644 (PMC7004307; doi:10.1371/journal.pone.0228644)
Supplement: S1 Table — (PDF) [file pone.0228644.s010.pdf]

Table S1. Calibration parameters of the linear regression for the quantification of DCF via fluorescence spectroscopy.

Working range [nM]	a [μV/nM]	b [μV]	LOD [nM]	LOQ [nM]
1 - 10	148.6	-17.1	0.03	0.1
10 - 150	149.1	-22.1	1.21	3.6
150 - 1000	143.7	558.0	9.85	29.5

a: slope, b: intercept, LOD: limit of detection, LOQ: limit of quantification
