# Supplementary material for: Pyrocatalysis—The DCF assay as a pH-robust tool to determine the oxidation capability of thermally excited pyroelectric powders
Source: PLoS One. 2020 Feb 6;15(2):e0228644. doi: 10.1371/journal.pone.0228644 (PMC7004307; doi:10.1371/journal.pone.0228644)
Supplement: S2 Table — (PDF) [file pone.0228644.s011.pdf]

Table S2. Crystallographic data of Rietveld refinement for BT, LN and LT powders.

Sample	BT		LN	LT	
RWP [%]	10.8		10.7	8.3	
goodness of fit	1.26		1.82	1.96	
compounds	$BaTiO_3$	$BaTiO_3$	$LiNbO_3$	$LiTaO_3$	Ta_2O_5
crystal system	cubic	tetragonal	trigonal	trigonal	orthorhombic
space group	$Pm\bar{3}m$	*P4mm*	*R3c*	*R3c*	*Pccm*
lattice parameters					
a [Å]	4.0089(2)	3.9946(1)	5.1504(1)	5.1508(1)	6.1934(9)
b [Å]	4.0089(2)	3.9946(1)	5.1504(1)	5.1508(1)	3.6614(5)
c [Å]	4.0089(2)	4.0329(1)	13.8636(2)	13.7684(2)	7.7818(9)
weight fraction [%]	25.0 ± 1.1	75.0 ± 1.1	100	89.1 ± 0.1	10.9 ± 0.1

BT: $BaTiO_3$, LN: $LiNbO_3$, LT: $LiTaO_3$
